# Evaluation of occupational health and safety intervention for the waste and sanitation workers in Bangladesh during COVID-19

**DOI:** 10.1016/j.ijheh.2023.114288

**Published:** 2024-01

**Authors:** Dewan Muhammad Shoaib, Tanvir Ahmed, Kazy Farhat Tabassum, Mehedi Hasan, Fazle Sharior, Mahbubur Rahman, Makfie Farah, Md Azizur Rahman, Alauddin Ahmed, James B. Tidwell, Mahbub-Ul Alam

**Affiliations:** a Environmental Health and WASH, Health System and Population Studies Division, International Center for Diarrhoeal Disease Research, Bangladesh (icddr,b), Dhaka, 1212, Bangladesh; b ITN-BUET Centre for Water Supply and Waste Management, BUET, Dhaka, 1000, Bangladesh; c Department of Civil Engineering, BUET, Dhaka, 1000, Bangladesh; d Harvard Kennedy School of Government, Cambridge, MA, 02138, USA; e World Vision, Inc., Washington, DC, 20002, USA; f School of Civil Engineering, University of Leeds, Leeds, LS2 9JT, United Kingdom

**Keywords:** Evaluation, Waste and sanitation workers, Occupational health and safety, Use of PPE, COVID-19

## Abstract

Waste and sanitation workers in South-Asian countries are vulnerable to injuries and diseases, including COVID-19. In Bangladesh, an intervention was implemented during COVID-19 to lower these workers' occupational health risks through training and PPE distribution.

We assessed how the intervention affected their occupational health behaviors using a randomized cluster trial in 10 Bangladeshi cities, including seven intervention and three control areas. We conducted 499 surveys (Control-152, Intervention-347) and 47 structured observations (Control:15, Intervention:32) at baseline and 499 surveys (Control:150, Intervention:349) and 50 structured observations (Control:15, Intervention:35) at endline. To evaluate the impact of intervention at the endline, we used the difference in difference (DID) method.

Compared to control, workers from intervention areas were more likely to have increased knowledge of using/maintaining PPEs (adjusted DID: 21%, CI: 8, 33), major COVID-19 transmission causes (adjusted DID: 27%, CI: 14, 40), and preventive measures (adjusted DID: 12%, CI: -0.6, 25), and improved attitude about using PPEs (adjusted DID: 36%, CI: 22, 49), washing PPEs (adjusted DID: 20%, CI: 8, 32). The intervention was more likely to improve workers' self-reported practice of taking adequate precautions after getting back from work (adjusted DID: 37%, CI: 27, 47) and changing/cleaning masks every day (adjusted DID: 47%, CI: 0.03, 94), and observed practices of maintaining coughing etiquette (DID: 20%, CI: 0.2, 40) at workplace and handwashing before wearing PPEs (DID: 27%, CI: 2, 52), after finishing work (DID: 31%, CI: -7, 69) & during work intervals (DID: 30%, CI: -33, 93). There was not much improvement in observed practices of mask use (DID: 1%, CI: -40, 42), handwashing before meals (DID: 2%, CI: -61, 65), and after toilet (DID: 7%, CI: -41, 55).

This intervention has improved the knowledge, attitude and practice of the workers about critical occupational risk mitigation, which may be replicated in similar settings. Future interventions need to address occupational health-related injuries and health complications, introduce regular health checkups/insurance for the workers, create a balance between the quality and comfort of the PPEs and ensure a mechanism to ensure a regular supply of PPEs.

## 1 Introduction

The waste and sanitation workers in South Asian countries often face vulnerability to various occupational injuries and diseases and are financially and culturally marginalized (Islam et al., 2016, World Bank et al., 2019, WaterAid Bangladesh, 2020). Many workers live in overcrowded, resource-poor urban settings with unhygienic water and sanitation facilities, leading to diverse health problems (World Bank et al., 2019). They also come across various hazardous compounds and toxic gases at their workplace, putting them at risk for multiple health issues such as respiratory disorders, skin diseases, infectious diseases, musculoskeletal disorders, injuries, and even death (Center for Water and sanitation et al., 2020, Oza et al., 2022, SNV, 2017). Like many low-and-middle-income countries (LMICs), waste and sanitation workers in Bangladesh handle waste without using any personal protective equipment (PPEs), increasing their susceptibility to injuries and diseases (Black et al., 2019, World Bank et al., 2019, WaterAid Bangladesh, 2020). The workers lack formal training on occupational health and safety, have insufficient knowledge about their health risks, and rarely take adequate safety measures at the workplace (Asibey et al., 2019, Degavi et al., 2021, Sharior et al., 2023, World Bank et al., 2019).

The COVID-19 pandemic has become a burden for their existing occupational health situation. Although the waste and sanitation workers served as the frontline workers during the pandemic, they became the overlooked group for COVID-19-specific training, support, and protective equipment distribution (Hoque, 2020, Mahase, 2021, WaterAid, 2021). Approximately 40% of sanitation workers in South Asian countries, including Bangladesh, Nepal, India, and Pakistan, reported not having handwashing stations in their workplace, which resulted in inconsistent handwashing practices during COVID-19 (WaterAid, 2021, WaterAid Bangladesh, 2020). Around 33% of the waste workers in Nepal did not receive any PPEs during COVID-19 (WaterAid, 2021). In India, roughly two-thirds of sanitation workers have not received COVID-19 prevention-related instructions or training to ensure workplace safety (Nigam et al., 2020). Moreover, around 80% of waste workers perceive that they are at risk of getting infected with COVID-19 because of their job nature. Medical waste handlers are at the highest risk, with around 61% considering themselves highly susceptible to COVID-19 (WaterAid, 2020).

Bangladesh is one of the hotspots of COVID-19, with 1,699,964 positive tested cases and 28,238 reported deaths till 25 January 2022 (WHO, 2022). A previous study in Bangladesh showed that around 50% of the waste and sanitation workers and their family members experienced symptoms related to COVID-19 (Hoque, 2020). Since the waste and sanitation workers work in people's homes, highways, healthcare facilities, and other institutions, they are exposed to infection and the chance of spreading the disease through them is high (WaterAid Bangladesh, 2020). Inadequate PPEs with a lack of standard operating guidelines, the lack of hygiene knowledge and occupational health safety training, and insufficient hygiene facilities at the workplace have long been the leading causes of the high burden of infections among workers (FANSAWSSCC, 2015, Hoque, 2020, WaterAid, 2020, World Bank et al., 2019, Zaqout et al., 2020). Therefore, it is necessary to ensure the safety of waste and sanitation workers and their families to mitigate the risk of spreading any infection, especially COVID-19 (WaterAid, 2021, WaterAid Bangladesh, 2020).

To address the issue and ensure the occupational health and safety of the workers, the Department of Public Health Engineering Bangladesh, in collaboration with the International Training Network Centre of Bangladesh University of Engineering and Technology (ITN-BUET), provided a capacity-building intervention considering their occupational health and safety (OHS), and infection prevention and control (IPC). The intervention included PPE delivery to the workers to ensure PPE use and training to enhance their personal and occupational safety management behaviour.

While the high risk of infection and diseases among waste and sanitation workers and the recommendations for interventions have been identified through several studies in different LMIC settings (Alam et al., 2022, Oza et al., 2022, Ramitha, 2023, Sharior et al., 2023, Ye et al., 2022), very few studies looked at evaluating the specific interventions to see whether the interventions work and in which areas they work and where they do not. Few studies that evaluated interventions were specifically focused on medical waste handlers in hospital settings (Elnour et al., 2015, Hosny et al., 2018, Tabash et al., 2016) and largely missed other types of waste and sanitation workers. In Egypt, one such intervention (including education and skill training) was evaluated, which improved waste management-related knowledge attitude and practice of the medical waste handlers (Hosny et al., 2018). In India, another educational intervention significantly improved the waste management knowledge of nursing and sanitation staff (Elnour et al., 2015). During COVID-19, the Bangladesh government took several initiatives (including providing safety equipment, infrastructure repairment, instructions on adhering to the guidelines, and monitoring of the waste management system) to ensure OHS of the waste and sanitation workers across all 64 districts of Bangladesh (Department of Public Health Engineering) but we do not know how well those initiatives worked. This scenario is also true for most of the LMICs where we do not know whether the interventions (both before and during COVID-19) are adequate to ensure the OHS of the workers and where we need more attention. Evaluating the intervention would allow us to understand the areas that need to be improved and tailor the intervention based on evidence, which would ultimately help in reducing intervention delivery costs, and improve the sustainability of the intervention. Thus, the objective of our study was to evaluate the effect of the intervention on occupational health safety-related knowledge, attitude, and practice of the waste and sanitation workers in selected study sites.

## 2 Material and methods

### 2.1 Study design and site selection

We conducted a randomized cluster trial with a baseline study (Alam, 2021, Alam et al., 2022) before and an endline study after the intervention in 10 cities of Bangladesh that included three city corporations and seven municipalities. A city corporation is an autonomous governing entity of a local government structure connected with the state government through the district administration. A municipality is an administrative body of local government directly connected with and governed by the state government. Bangladesh has 12 city corporations and 329 municipalities (Star Online Report, 2018, LGED. About LGIs, 2018). Among the ten cities that we selected, seven were intervention areas with two City Corporations (Mymensingh City Corporation and Cumilla City Corporation) and five Municipalities (Satkhira Municipality, Laksham Municipality, Cox's Bazar Municipality, Moulvibazar Municipality, and Bhola Municipality), and three were control areas with one City Corporation (Rangpur City Corporation) and two Municipalities (Sirajganj Municipality, and Manikgonj Municipality). We selected the seven intervention areas purposively from the 14 areas (3 City Corporations and 11 Municipalities) where ITN-BUET, CWIS-FSM support sell, and Department of Public Health Engineering (DPHE) provided the intervention considering the diversity of geographical locations. The three control areas were selected purposively considering the similarities in socio-economical and geographic characteristics of much of the intervention areas. The selected areas also ensured representation from all the divisions of Bangladesh (Table 1).

**Table 1:** Study areas based on control and intervention group.

Group	Areas	Areas that received the intervention	Purposively Selected study areas
Intervention Areas	City Corporation	1. Mymensingh City Corporation 2. Rangpur City Corporation 3. Comilla City Corporation	1. Mymensingh City Corporation 2. Comilla City Corporation
	Municipalities	4. Lakhsmipur Municipality 5. Satkhira Municipality 6. Mongla Municipality 7. Cox's Bazar Municipality 8. Moulvibazar Municipality 9. Munshiganj Municipality 10. Bhola Municipality 11. Patuakhali Municipality 12. Laksam Municipality 13. Ishwardi Municipality 14. Sakhipur Municipality	3. Satkhira Municipality 4. Cox's Bazar Municipality 5. Moulvibazar Municipality 6. Bhola Municipality 7. Laksam Municipality
Control Areas	City Corporation	N/A	8. Rangpur City Corporation
	Municipalities	N/A	9. Sirajganj Municipality 10. Manikgonj Municipality

### 2.2 Population group selection

We reached five types of frontline waste and sanitation workers in the study: septic tank/pit emptiers, solid waste collectors, drain cleaners, road sweepers, and medical waste handlers. As the workers were involved in more than one job at a time (e.g., the pit emptier was also engaged in drain cleaning), we categorized them based on their level of involvement. For instance, we selected them as road sweepers if the workers were primarily involved in sweeping while other tasks were secondary. We reached both listed workers (listed in the city offices as waste and sanitation workers) and non-listed workers (working individually/informally/for private organizations) from both control and intervention areas.

### 2.3 Sample size calculation

We estimated the sample size based on the prevalence of 22% of the waste and sanitation workers who did not maintain hygiene practices at their workplaces and expected a 12% improvement in hygiene practices (WaterAid, 2021) among the workers as an effect of the intervention compared to the control areas. To estimate the required sample size, a two-independent sample test of equity of proportions (Chow et al., 2017) was carried out with the consideration of a ratio of 0.4 of the control group size to the intervention group size since, in our study, 7 of the ten localities were intervention regions and 3 were control areas. Therefore, according to the sample size calculation, we need 339 workers from the intervention areas and 136 from the control areas after adjusting for the 15% non-response rate. Thus, a total sample of 475 workers was needed for both groups to achieve 80% power at a 0.05 significance level.

### 2.4 Baseline and endline data collection

The field enumerators collected data at baseline (pre-intervention) and endline (post-intervention) through surveys and structured observations in both intervention and control areas. Before initiating the data collection, the field enumerators collected the lists of waste and sanitation workers officially enlisted under the City Corporation and Municipalities in the selected study areas. Then, they estimated the total number of workers who were not enlisted (informal workers) but were working at the frontline as waste and sanitation workers by discussing with respective authorities and the enlisted workers. After that, they compared it with the number of listed workers and reached the participants based on population proportion to size (listed vs. non-listed) in each area. The listed workers were selected randomly from the list collected from city offices for the survey, and the non-listed workers were selected using snowball sampling. The observation participants were selected purposively from the survey participants, considering the diversity of the age-gender group and work type.

The field enumerators conducted face-to-face interviews and structured observations. For the observation, the enumerators accompanied/reached the workers at their workplace and stayed with them during work. Field enumerators collected the baseline data from 20 October 2020 to 15 November 2020 and the endline data from 30 June 2021 to 05 August 2021, roughly after one month of intervention provision.

### *2.5* *Intervention* delivery

The intervention reached the selected cities' workers from December 2020 to May 2021. Thirty-five master trainers (experts in waste management program and research, WASH, and behavior change professionals) were recruited, who organized a two-day online training of trainers (ToT) sessions (4 hours a day) for the 381 city-level trainers (city officials involved in waste and sanitation management in the city corporations and municipalities) from each intervention area. After completion of ToT, ITN-BUET delivered the intervention to 4900 frontline waste and sanitation workers through the city officials that received the ToT. The intervention contained a 4-hour face-to-face training session, PPE distribution involving DPHE, and a practical demonstration of properly using PPEs. The training contained topics on how infectious diseases (especially COVID-19) spread and the necessary measures to tackle them. A particular emphasis is given on handwashing, how to disinfect themselves and their equipment after work, what the workers can do if they get infected with infectious diseases, and how to use PPEs with practical demonstration and practice about properly wearing them (ITN-BUET, 2021). Each training participant received a guideline (developed with pictures and visualizations to make it easily understandable) on maintaining OHS and IPC (ITN-BUET, 2020). The PPEs delivered to the workers (face masks, hand gloves, goggles, safety shoes, safety gumboot, safety helmet, apron, and reflective jacket) were considered based on their work types. For instance, drain cleaners received only masks, gloves, goggles, safety gumboots, and reflective jackets as they needed these PPEs only.

### 2.6 Outcome variable

The primary outcome variable of this study is the practice of the workers in using and cleaning PPEs and masks. The secondary outcome variables included the workers' knowledge, attitude and practice regarding key COVID-19 preventive and workplace safety measures.

### 2.7 Data analysis

Descriptive statistics of the waste and sanitation workers' socio-demographic characteristics were computed for the baseline, endline period, and their underlying intervention and control group and summarized in tables comparing study arms. Then, a balance test is conducted to assess for any differences in the socio-demographic characteristics of the respondents between the treatment and control groups at baseline. We calculated proportions for categorical variables, means for symmetric continuous variables and medians for skewed continuous variables to test for these differences. Then, we performed a chi-squared test of homogeneity to compare the difference of proportions for categorical variables, a two-sample *t*-test was performed to test the difference in means for continuous variables, and quantile regression was used to test the equality of medians between the intervention and control group. To evaluate the impact of the intervention at the endline, we used the difference in difference (DID) method. We assessed the parallel trend assumption through visual inspection of the line graph for the intervention and control group over the pre and post-intervention period, which illustrated no time-varying difference between the intervention and control groups. We used a logistic regression model to assess the impact of the intervention on the binary outcome measures. And we fit a generalized linear model with an identity link to obtain the DID estimates for those variables exhibiting separation and multicollinearity problems. The average marginal effects of these models are reported for ease of interpretation. DID estimates with 95% confidence intervals (CI) to test post-versus pre-intervention implementation changes between the intervention and control groups. We have adjusted the regression models with the covariate's education, religion, caste, accommodation, and monthly income of the respondents, which showed a significant difference at baseline between intervention and control groups. All the variables are considered statistically significant at p < 0.5.

### 2.8 Ethical *considerations*

The study participants went through an informed consent process. During data collection, our field staff maintained the government-guided COVID-19 prevention measures (e.g., mask use and social distancing). The study protocol received ethical approval from the ITN-BUET ethical review panel.

## 3 Results

### 3.1 Socio-demographic characteristics of the respondents

The data collectors conducted 499 surveys (Control:152; Intervention:347) and 47 structured observations (Control:15; Intervention:32) in baseline, 499 surveys (Control:150; Intervention:349), and 50 structured observations (Control:15; Intervention:35) in endline with the five types of frontline waste and sanitation workers.

Participants from the intervention and control areas in both baseline and endline had similar socio-demographic characteristics regarding gender, age, religion, education, income, and accommodation facilities. Female workers were mostly involved only in road sweeping; thus, most of the workers we reached were male. Around half were Muslims, and the remaining half were Hindus (mostly Harijan/Dalit caste). We found the highest numbers of workers between the ages of 20 and 40. Most workers could write their names or have completed only primary education. They mostly lived in rented houses or government-provided accommodation facilities with an average monthly income (individual) of 7000–9000 BDT (82–105 USD). The baseline status of the respondents in receiving OHS training, availability of safety guidelines, and training on PPE were also similar in intervention and control areas, which then improved in the intervention areas at the endline **(**Table 2**)**.

**Table 2:** Socio-demographic characteristics of waste and sanitation workers.

Indicators	Baseline			Endline		
	Intervention n (%) N = 347	Control n (%) N = 152	Difference (p-value)	Intervention n (%) N = 349	Control n (%) N = 150	Difference (p-value)
Work type						
Septic tank/Pit-emptier	51 (15)	25 (16)	−1 (0.617)	80 (23)	31 (21)	2 (0.578)
Solid waste collector	118 (34)	55 (36)	−2 (0.638)	142 (41)	52 (35)	6 (0.206)
Drain cleaner	89 (26)	40 (26)	0 (0.875)	123 (35)	39 (26)	9 (0.043)
Road sweeper	131 (38)	43 (28)	10 (0.041)	138 (40)	36 (24)	16 (0.001)
Medical waste handler	58 (17)	20 (13)	4 (0.314)	58 (17)	30 (20)	−3 (0.364)
**Respondent's gender**						
Male	285 (82)	118 (78)	4 (0.240)	279 (80)	125 (83)	3 (0.376)
Female	62 (18)	34 (22)	−4	70 (20)	25 (17)	3
**Respondent's age**						
<20	15 (4)	14 (9)	−5 (0.086)	15 (4)	7 (5)	−1 (0.341)
20–30	136 (39)	48 (32)	7	144 (41)	47 (31)	10
31–40	103 (30)	39 (26)	4	97 (28)	51 (34)	−6
41–50	58 (17)	30 (20)	−3	45 (13)	26 (17)	−4
51–60	22 (6)	16 (10)	−4	32 (9)	13 (9)	0
>60	13 (4)	5 (3)	1	16 (5)	6 (4)	1
**Respondent's age**						
Mean (±SD)	35 (12.18)	37 (13.37)	−1.2 (0.242)	35 (12.23)	37 (12.39)	0 (0.112)
**Respondent's religion**						
Islam	186 (54)	64 (42)	12 (0.014)	192 (55)	75 (50)	5 (0.530)
Hinduism	161 (46)	87 (57)	−11	156 (45)	75 (50)	−5
Other	0 (0)	1 (0.66)	−1	1 (0.29)	0 (0)	0.3
**Respondent's caste**						
Harijan/Dalit	123 (35)	85 (56)	−21 (0.000)	139 (40)	74 (49)	−9 (0.049)
Others	224 (65)	67 (44)	21	210 (60)	76 (51)	9
**Respondent's Education**						
No formal education	50 (14)	31 (20)	−6 (0.001)	64 (18)	19 (13)	5 (0.000)
Can sign only	128 (37)	30 (20)	17	129 (37)	77 (51)	−14
1-8th class	150 (43)	76 (50)	−7	146 (42)	38 (25)	17
Above 8th class	19 (5.48)	15 (9.87)	−4	10 (2.87)	16 (11)	−8
**Household members**						
less or equal 2 members	9 (2)	10 (6)	−4 (0.197)	12 (4)	7 (5)	−1 (0.259)
3-5 members	202 (58)	83 (55)	3	210 (60)	98 (65)	−5
6-7 members	89 (26)	38 (25)	1	81 (23)	34 (23)	0
More than 7 members	47 (14)	21 (14)	0	46 (13)	11 (7)	6
**Accommodation**						
Government facilities	122 (35)	85 (56)	−21(0.000)	126 (36)	53 (35)	1(0.000)
Non-Government facilities	3 (1)	1 (1)	0	10 (3)	0 (0)	3
Own facilities	68 (20)	42 (27)	−7	67 (19)	56 (37)	−18
Rent	153 (44)	21 (14)	30	128 (37)	40 (27)	10
Other	1 (0.29)	3 (2)	−2	18 (5)	1 (1)	4
**Monthly income (BDT)**						
Mean (±SD)	8881 (4847.83)	7179 (5918.91)	−1071 (0.002)	7966 (4768.15)	7143 (4633.87)	134 (0.033)
**Workers trained in occupational health safety**	70 (20)	29 (19)	1 (0.778)	305 (87)	19 (13)	74 (0.000)
**Workers trained on PPE use**	51 (15)	21 (14)	1 (0.796)	305 (87)	3 (2)	85 (0.000)

### 3.2 Effect on the knowledge and attitude of the workers

Compared to the control areas, the workers from intervention areas were more likely to have improved knowledge regarding the availability of occupational health and safety guidelines (adjusted DID: 57%, CI: 44, 69), the importance of using and maintaining protective equipment (adjusted DID: 21%, CI: 8, 33), the concept of communicable diseases (adjusted DID: 13%, CI: 0, 26), the significant causes of COVID-19 transmission (adjusted DID: 27%, CI: 14, 40), and key COVID-19 preventive measures (adjusted DID: 12%, CI: −0.6, 25). A particular positive association is seen between the intervention and the improved knowledge in the intervention areas on used utensils (adjusted DID: 22%, CI: 12, 32) and social gathering (adjusted DID: 24%, CI: 11, 37) as means of COVID-19 transmission, and using goggles (adjusted DID: 10%, CI: 2, 18) and maintaining social distancing (adjusted DID: 22%, CI: 9, 35) as key COVID-19 preventive measures. Despite showing improvement in knowledge among the workers in the endline intervention areas, understanding coughing (adjusted DID: −12%, CI: −21, −3), sneezing (adjusted DID: −3%, CI: −14, 8), and spittle (adjusted DID: 5%, CI: −7, 17) as major COVID-19 spread mechanisms, and knowledge to avoid touching nose, eyes, and mouth with unclean hands (adjusted DID: −1%, CI: −13, 11), maintaining coughing etiquette (adjusted DID: −11%, CI: −20, −2), and vaccination (adjusted DID: 2%, CI: −2, 6) as important COVID-19 prevention measure are less likely to be associated with the intervention provision because a similar change is also seen in endline control areas. Workers’ understanding about considering air (adjusted DID: −24%, CI: −36, −12) and water (adjusted DID: −22%, CI: −36, −8) as means to spread communicable to did not improve while their knowledge of considering touch as a means to spread communicable diseases improved (adjusted DID: 16%, CI: −1, 33) (although this was not statistically significant). Moreover, the intervention seems to have no effect in changing the workers' knowledge of wearing face masks, gloves, and handwashing as COVID-19 preventive measures. Similarly, the slight improvements in knowledge of the workers from the intervention areas in properly understanding the terms social distancing (adjusted DID: 4%, CI: −8, 16), quarantine (adjusted DID: 3%, CI: −20, 26), and isolation (adjusted DID: 5%, CI: −31, 41) than the workers from control areas seems to have less association with the intervention.

Workers' improved attitude towards using PPEs (adjusted DID: 36%, CI: 22, 49), washing PPEs (adjusted DID: 20%, CI: 8, 32), cleaning working equipment regularly (adjusted DID: 20%, CI: 7, 33), and keeping open sores, cuts, and wounds covered with clean, dry band-aids (adjusted DID: 13%, CI: 2, 24) can be attributed to the impact of the intervention. Their attitude towards handwashing before and after using toilets (adjusted DID: 9%, CI: −3, 22) and taking meals (adjusted DID: −4%, CI: −18, 9) seems to be less associated with the intervention. The intervention also did not improve workers’ perception towards considering cleaning equipment using anti-germ products (adjusted DID: −7%, CI: −15, 0.7). On the other hand, a significantly improved percentage of workers at endline intervention areas reported that they believe OHS interventions can reduce health risks at the workplace (adjusted DID: 58%, CI: 26, 89), which is more likely to be contributed as a resulting factor of the intervention **(**Table 3**)**

**Table 3:** Knowledge and attitude of the workers on maintaining occupational health and safety.

Indicators	Intervention			Control			DID (95% CI)	Adjusted DID (95% CI)
	Baseline n (%) N = 347	End line n (%) N = 349	Difference (95% CI)	Baseline n (%) N = 152	End line n (%) N = 150	Difference (95% CI)		
**Workers know about the availability of occupational health safety guidelines**	104 (30)	264 (76)	45 (39, 51)	63 (41)	44 (29)	−12 (−23, −1)	**57 (45, 69)**	**57 (44, 69)**
**Know about the importance of using and maintaining PPEs**	211 (61)	323 (93)	32 (26, 38)	82 (54)	93 (62)	8 (−3, 19)	**24 (11, 37)**	**21 (8, 33)**
**Know about communicable diseases**	169 (49)	253 (73)	24 (17, 31)	82 (54)	97 (65)	11 (−0.2, 21)	**13 (0, 26)**	**16 (3, 29)**
**Know how communicable diseases spread**	**N=169**	**N=253**		**N=82**	**N=97**			
Through air	150 (89)	234 (92)	3 (−2, 8)	64 (78)	96 (99)	21 (12, 30)	**−17 (-28, -6)**	**−24 (-36, -12)**
Through water	71 (42)	116 (46)	4 (−5, 13)	7 (8.54)	44 (45)	36 (25, 48)	**−32 (-48, -16)**	**−22 (-36, -8)**
Through touch	105 (62)	205 (81)	19 (10, 28)	49 (60)	63 (65)	5 (−9, 19)	14 (−3, 31)	16 (−1, 33)
Through vectors	21 (12)	22 (8.7)	−3 (−9, 2)	2 (2.44)	1 (1.03)	−1 (−5, 2)	−2 (−9, 5)	−3 (−10, 4)
Don't know	2 (1.18)	0 (0)	−1 (−4, 2)	16 (20)	2 (2.06)	−17 (−22, −12)	**16 (10, 22)**	**16 (10, 22)**
**Workers' knowledge of how COVID-19 spread**								
Coughing	306 (88)	327 (94)	6 (1, 11)	118 (78)	143 (95)	18 (10, 25)	**−12 (-21, -3)**	**−12 (-21, -3)**
Sneezing	253 (73)	309 (89)	15 (9, 21)	113 (74)	132 (88)	13 (4, 22)	2 (−8, 12)	−3 (−14, 8)
Spittle	136 (39)	211 (60)	21 (14, 28)	30 (20)	58 (39)	19 (9, 29)	2 (−10, 14)	2 (−10, 14)
Used utensils	73 (21)	127 (36)	15 (8, 22)	25 (16)	12 (8)	−8 (−15, −1)	**23 (13, 33)**	**22 (12, 32)**
Social gathering	194 (56)	197 (56)	0 (−7, 7)	81 (53)	47 (31)	−22 (−33, −11)	**22 (9, 35)**	**24 (11, 37)**
**Workers have key knowledge of ways of COVID-19 transmission (considered coughing, sneezing, and social gathering)**	142 (41)	172 (49)	8 (1, 15)	70 (46)	38 (25)	−21 (−31, −11)	**29 (16, 42)**	**27 (14, 40)**
**Worker's knowledge of how to prevent COVID-19**								
Wearing face mask	325 (94)	331 (95)	1 (−2, 4)	138 (91)	143 (95)	4 (−1, 10)	−3 (−10, 3)	−3 (−11, 5)
Goggles	13 (3.75)	65 (19)	15 (10,20)	7 (4.61)	18 (12)	7 (1, 13)	7 (−0.1, 15)	**10 (2, 18)**
Using hand gloves	148 (43)	151 (43)	0 (−7, 7)	44 (29)	48 (32)	3 (−7, 13)	−2 (−15, 10)	−2 (−14, 10)
Hand washing	253 (73)	256 (73)	0 (−6, 6)	97 (64)	114 (76)	12 (2, 22)	−12 (−24, 0.4)	−11 (−23, 1)
Not touching the eye, nose, or face with unclean hands	81 (23)	129 (37)	14 (7, 21)	40 (26)	59 (39)	13 (3, 23)	0.6 (−11, 13)	−1 (−13, 11)
Maintaining social distance	194 (56)	255 (73)	17 (10, 24)	81 (53)	75 (50)	−3 (−14, 8)	**20 (7, 33)**	**22 (9, 35)**
Washing cloths	67 (19)	86 (25)	5 (−0.8, 11)	36 (24)	31 (21)	−3 (−12, 6)	8 (−2, 19)	7 (−4, 18)
Cleaning fruits and vegetables	24 (6.92)	43 (12)	5 (1, 9)	8 (5.26)	21 (14)	8 (2, 15)	−3 (−11,4)	−1 (−9, 7)
Using the elbow while coughing or sneezing	31 (8.93)	21 (6.02)	−3 (−7, 1)	20 (13)	34 (23)	9 (1, 17)	**−12 (-21, -3)**	**−11 (-20, -2)**
Cleaning toilets	17 (4.9)	3 (0.86)	−4 (−6, −2)	5 (3.29)	12 (8)	4 (−0.4, 9)	**−8 (-15, -3)**	**−8 (-13, -3)**
Washing shoes or other equipment	14 (4.03)	5 (1.43)	−2 (−5, −0.1)	11 (7.24)	7 (4.67)	−2 (−7, 3)	0 (−5, 5)	−1 (−6, 4)
Medicine	5 (1.44)	10 (2.87)	1 (−0.7, 3)	3 (1.97)	2 (1.33)	−0.6 (−3, 2)	2 (−1, 5)	3 (−1, 7)
Vaccination	2 (0.58)	20 (5.73)	5 (3, 7)	0 (0)	4 (2.67)	3 (−0.9, 7)	2 (−2, 6)	2 (−2, 6)
**Workers have key knowledge on preventing COVID-19 transmission (Considered wearing a face mask, Hand washing, and Maintaining social distancing)**	145 (42)	200 (57)	15 (8, 22)	55 (36)	63 (42)	5 (−5, 15)	10 (−3, 23)	12 (−0.6, 25)
**Worker's knowledge of when to wear a face mask**								
When we go out of home	312 (90)	336 (96)	6 (2, 10)	142 (93)	146 (97)	4 (−0.7, 8)	2 (−3, 8)	3 (−3, 9)
When we meet anyone	90 (26)	186 (53)	27 (20, 34)	34 (22)	62 (41)	19 (8, 29)	8 (−4, 20)	4 (−8, 16)
When caring the patients	30 (8.65)	88 (25)	16 (11, 22)	9 (5.92)	22 (15)	8 (1, 15)	8 (−1, 17)	7 (−1, 15)
When we feel sick/cough/sneeze	45 (13)	105 (30)	17 (11, 23)	10 (6.58)	47 (31)	24 (16, 33)	−7 (−17, 3)	−7 (−17, 3)
When we handle waste	188 (54)	216 (62)	8 (0.4, 15)	67 (44)	90 (60)	16 (5, 27)	−8 (−21, 5)	−11 (−24, 2)
When we can't maintain social distance anyway	45 (13)	112 (32)	19 (13, 25)	6 (3.95)	37 (25)	20 (13, 28)	−1 (−11, 8)	−1 (−10, 8)
**Correctly mentioned social distancing**	219 (63)	275 (86)	23 (16, 29)	99 (65)	84 (82)	17 (6, 27)	6 (−6, 18)	4 (−8, 16)
**Correctly mentioned quarantine**	85 (78)	84 (50)	−28 (−38, −18)	23 (62)	10 (21)	−41 (−60, −21)	13 (−8, 35)	3 (−20, 26)
**Correctly mentioned isolation**	24 (59)	22 (63)	4 (−17, 26)	16 (55)	9 (56)	1 (−29, 31)	3 (−34, 40)	5 (−31, 41)
**Opinion on health & hygiene precautions needed at work**								
Washing hands before and after the toilet	196 (56)	235 (67)	11 (4, 18)	99 (65)	100 (67)	2 (−9, 13)	9 (−4, 22)	9 (−3, 22)
Use PPE	179 (52)	271 (78)	26 (19, 33)	88 (58)	68 (45)	−13 (−24, −2)	**39 (26, 52)**	**36 (22, 49)**
Washing PPE	90 (26)	187 (54)	27 (20, 34)	29 (19)	38 (25)	6 (−3, 15)	**21 (9, 33)**	**20 (8, 32)**
Cleaning working tools	221 (64)	239 (68)	4 (−3, 11)	85 (56)	62 (41)	−15 (−26, −4)	**19 (6, 32)**	**20 (7, 33)**
Avoid touching face, eye, or mouth with unclean hands	146 (42)	211 (60)	18 (11, 25)	66 (43)	103 (69)	25 (14, 36)	−7 (−20, 6)	−6 (−19, 7)
Washing hands with soap and water before eating or drinking.	177 (51)	187 (54)	3 (−5, 1)	73 (48)	88 (59)	11 (−0.5, 21)	−8 (−21, 5)	−4 (−18, 9)
Keep open sores, cuts, and wounds covered with a clean, dry band-aid.	47 (14)	74 (21)	7 (1, 13)	38 (25)	29 (19)	−6 (−15, 3)	**13 (2, 24)**	**13 (2, 24)**
**Consider it necessary to clean the working equipment with anti-germ products**	312 (90)	337 (97)	7 (3, 11)	124 (82)	146 (97)	15 (8, 22)	**−9 (-17, -1)**	−7 (−15, 0.7)
**Benefits of OHS training**	**N=70**	**N=305**		**N=29**	**N=19**			
Operation & maintenance	40 (57)	300 (98)	41 (29, 53)	19 (66)	17 (89)	23 (1, 45)	18 (−7, 41)	17 (−6, 39)
Use of PPEs	23 (33)	301 (99)	66 (54, 78)	11 (38)	8 (42)	4 (−24, 32)	**62 (31, 93)**	**56 (25, 88)**
Safety measures	38 (54)	300 (98)	44 (32, 56)	22 (76)	16 (84)	8 (−16, 32)	**36 (10, 62)**	**34 (14, 54)**
Reduce health risk	34 (49)	301 (99)	50 (38, 62)	18 (62)	11 (58)	−4 (−32, 24)	**54 (23, 85)**	**58 (26, 89)**
Capacity building	23 (33)	81 (27)	−6 (−18, 6)	17 (59)	9 (47)	−11 (−39, 17)	5 (−26, 36)	14 (−16, 45)
Increase knowledge	30 (43)	111 (36)	−7 (−19, 5)	15 (52)	10 (53)	1 (−28, 30)	−8 (−40, 24)	4 (−26, 34)
Increase confidence	7 (10)	60 (20)	10 (2, 18)	7 (24)	11 (58)	34 (7, 61)	−24 (−52, 4)	−22 (−51, 7)
Learn about infectious diseases	10 (14)	108 (35)	21 (11, 31)	1 (3)	4 (21)	18 (−1, 37)	3 (−18, 24)	1 (−21, 24)

### 3.3 Effect on workers' reported practice of maintaining OHS measures

#### 3.3.1 Reported practice

A noticeable improvement was seen in the practice of taking adequate precautions by the workers after returning home from work (adjusted DID: 37%, CI: 27, 47), which seems to have resulted from the intervention. Necessary precautions like keeping distance from family members before cleaning (adjusted DID: 22%, CI: 9, 35), washing hands first (adjusted DID: 39%, CI: 26, 52), and washing clothes (adjusted DID: 31%, CI: 18, 46) after returning home appear to be more associated with the intervention. The availability of PPEs was highly improved in intervention areas after the intervention provision. However, the use of PPEs among the workers who had PPEs available such as face masks (adjusted DID: −1%, CI: −10, 8), hand gloves (adjusted DID: −4%, CI: −17, 9), gumboot (adjusted DID: −1%, CI: −13, 11), and apron (adjusted DID: −6%, CI: −38, 26) stayed similar in both intervention areas and control areas. The common problems with PPEs that the workers faced were suffocation, heat and sweat, and unfit sizes. The intervention did not significantly affect their proper PPE disposal practices (adjusted DID: 2%, CI: −16, 20). Workers' reported improved practices of changing/cleaning masks every day (adjusted DID: 47%, CI: 0.03, 94), cleaning working equipment regularly (adjusted DID: 26%, CI: 17, 35), wearing PPEs while cleaning waste management vehicles (adjusted DID: 26%, CI: 17, 35) appear to be highly associated with the intervention (Table 4).

**Table 4:** Reported practice of the workers in maintaining OHS and COVID-19 prevention measures.

Indicators	Intervention			Control			DID (95% CI)	Adjusted DID (95% CI)
	Baseline n (%) N = 347	End line n (%) N = 349	Difference (95% CI)	Baseline n (%) N = 152	End line n (%) N = 150	Difference (95% CI)		
	N = 104	N = 264		N = 63	N = 44			
**Maintain OHS guidelines at work**	56 (54)	212 (80)	26 (15, 37)	49 (78)	37 (84)	6 (−9, 21)	20 (2, 38)	3 (−16, 22)
**Health and safety precaution taken by the workers during COVID-19 after returning home**								
Make distance from family	81 (23)	195 (56)	32 (25, 39)	54 (36)	69 (46)	10 (−0.5, 21)	**22 (9, 35)**	**22 (9, 35)**
Wash hands first	127 (37)	229 (66)	29 (22, 36)	65 (43)	45 (30)	−13 (−24, −2)	**42 (29, 55)**	**39 (26, 52)**
Wash cloth	170 (49)	258 (74)	25 (18, 32)	106 (70)	88 (59)	−11 (−21, −0.3)	**36 (23, 49)**	**31 (18, 46)**
Taking bath with soap	323 (93)	301 (86)	−7 (−12, −2)	143 (94)	127 (85)	−9 (−16, −2)	2 (−6, 10)	3 (−5, 11)
**Necessary precautions taken by the workers after returning home during COVID-19 (Considered keeping distance from family before cleaning, washing hand, cloth, and taking bath)**	26 (7.49)	108 (31)	23 (17, 29)	31 (20)	11 (7.33)	−13 (−21, −5)	**36 (27, 45)**	**37 (27, 47)**
**Availability of PPEs**	209 (60)	333 (95)	35 (30, 41)	73 (48)	77 (51)	3 (−8, 14)	**32 (19, 45)**	**26 (13, 38)**
**Types of PPEs available to the workers**	**N=209**	**N=333**		**N=73**	**N=77**			
Hand sanitizer	49 (23)	248 (74)	51 (44, 58)	13 (18)	13 (17)	−1 (−13, 11)	**52 (37, 67)**	**48 (35, 62)**
Face Mask	194 (93)	319 (96)	3 (−1, 7)	71 (97)	73 (95)	−2 (−8, 4)	5 (−2, 12)	5 (−1, 11)
Hand Gloves	153 (73)	260 (78)	5 (−3, 12)	42 (58)	18 (23)	−34 (−49, −19)	**39 (22, 56)**	**35 (18, 52)**
Face shield/protective glass	13 (6.22)	111 (33)	27 (21, 33)	4 (5.48)	0 (0)	−5 (−11, −1)	**32 (24, 40)**	**32 (19, 45)**
Gum boot	136 (65)	260 (78)	13 (5, 21)	40 (55)	24 (31)	−24 (−38, −8)	**37 (19, 55)**	**38 (22, 54)**
Half apron	32 (15)	142 (43)	27 (19, 35)	20 (27)	11 (14)	−13 (−27, 1)	**40 (24, 56)**	**39 (25, 53)**
Apron (clinical waste handler)	41 (20)	43 (13)	−7 (−13, −0.3)	6 (8.22)	12 (16)	7 (−4, 18)	**−14 (-27, -1)**	**−11 (-23, 1)**
Helmet	3 (1.44)	83 (25)	23 (18, 28)	6 (8.22)	2 (2.6)	−6 (−13, 1)	**29 (19, 39)**	**33 (23, 43)**
**Currently using any PPEs**	188 (90)	320 (96)	6 (1, 11)	68 (93)	76 (99)	6 (−0.7, 12)	0 (−7, 7)	0 (−9, 9)
**Current usage of different types of PPEs according to their availability**								
Hand sanitizer	N = 49 48 (98)	N = 248 245 (99)	1 (−3, 5)	N = 13 11 (85)	N = 13 13 (100)	15 (5, 25)	−**14 (-25, -3)**	**−15 (-25, -5)**
Face Mask	N = 194 176 (91)	N = 319 310 (97)	6 (2, 10)	N = 71 66 (93)	N = 73 72 (99)	5 (−1, 11)	1 (−7, 9)	−1 (−10, 8)
Hand Gloves	139 (91)	253 (97)	6 (2, 10)	38 (90)	18 (100)	9 (−3, 21)	−3 (−16, 10)	−4 (−17, 9)
Face shield/protective glass	13 (100)	11 (100)	0	4 (100)	–			
Gumboot	122 (90)	250 (96)	6 (1, 11)	36 (90)	23 (96)	6 (−6, 18)	0.6 (−13, 13)	−1 (−13, 11)
Half apron	30 (94)	137 (96)	2 (−6, 10)	18 (90)	11 (100)	10 (−5, 25)	−8 (−25, 9)	−7 (−24, 10)
Apron (clinical waste handler)	35 (85)	42 (98)	12 (0.5, 24)	5 (83)	11 (92)	8 (−26, 42)	4 (−31, 39)	−6 (−38, 26)
Helmet	3 (100)	81 (98)	−2 (−19, 15)	6 (100)	2 (100)	0 (−23, 23)	−2 (−31, 27)	2 (−27, 31)
Regular shoes	–	88 (97)		–				
Gas detectors	–	2 (100)		–				
Reflective jacket	–	99 (93)		–				
	**N=209**	**N=333**		**N=73**	**N=77**			
**Feel problem with PPE**	104 (50)	77 (23)	−27 (−35, −19)	38 (52)	16 (21)	−31 (−46, −16)	4 (−12, 20)	8 (−8, 24)
**Problem with PPEs**								
Doesn't fit	38 (18)	59 (18)	−0.4 (−6, 6)	19 (26)	1 (1.30)	−24 (−35, −15)	**24 (12, 36)**	**30 (17, 43)**
Suffocation	93 (45)	37 (11)	−34 (−41, −27)	35 (48)	7 (9)	−39 (−52, −26)	5 (−10, 20)	9 (−6, 24)
Heat & sweat	9 (4.31)	7 (2.10)	−2 (−5, 1)	3 (4.11)	9 (12)	8 (−1, 17)	**−10 (-19, -0.7)**	**−9 (-17, -1)**
**PPE disposal after use/management of damaged PPE**								
Burn to fire	19 (9.09)	49 (15)	5 (0.2, 11)	15 (21)	17 (22)	1 (−12, 14)	4 (−10, 18)	2 (−11, 15)
Throw it away	84 (40)	128 (38)	−2 (−10, 6)	60 (82)	55 (71)	−11 (−24, 2)	9 (−6, 24)	9 (−7, 26)
Throw in the respected bin	117 (56)	191 (57)	1 (−7, 9)	18 (25)	19 (25)	0.01 (−13, 13)	1 (−15, 17)	1 (−15, 17)
Can't remember	5 (2.39)	13 (3.9)	1 (−2, 4)	0 (0)	6 (7.79)	8 (2, 14)	−6 (−12, 0.03)	−4 (−10, 2)
Return to office	6 (2.87)	–	–	3 (4.11)	–	–		–
**Properly disposed PPEs (Considered burning and throwing in the respected bin)**	127 (61)	216 (65)	4 (−4, 12)	30 (41)	33 (43)	2 (−14, 18)	2 (−16, 20)	2 (−16, 20)
**Frequency of changing/cleaning mask (in days)**	**N=31**	**N=8**		**N=17**	**N=33**			
1 day	9 (29)	7 (88)	58 (30, 86)	11 (65)	23 (70)	5 (−22, 32)	**53 (14, 92)**	**47 (0.03, 94)**
2–3 days	13 (42)	1 (12)	−29 (−58, −0.6)	4 (23)	9 (27)	4 (−21, 29)	−33 (−71, 5)	−13 (−59, 33)
	**N=312**	**N=337**		**N=124**	**N=146**			
**Cleaning working equipment regularly**	288 (92)	322 (96)	3 (−0.4, 7)	120 (97)	104 (71)	−26 (−34, −18)	**29 (20, 38)**	**26 (17, 35)**
**Reasons for not cleaning working equipment and PPE**	**N=35**	**N=12**		**N=28**	**N=4**			
Use one-time PPE	2 (5.71)	1 (8.33)	3 (−6, 12)	1 (3.57)	1 (25)	21 (−7, 49)	−18 (−50, 14)	−43 (−110, 24)
Lack of cleaning agents	6 (17)	5 (42)	25	1 (3.57)	0 (0)	−4	**-**	**-**
High cost of cleaning	2 (5.71)	4 (33)	27	1 (3.57)	0 (0)	−4	**-**	**-**
No facility to wash	–	–	–	1 (3.57)	0 (0)	−4	**-**	**-**
Cleaning agent not provided by recruiter	16 (46)	4 (33)	−12 (−46, 22)	13 (46)	2 (50)	4 (−37, 45)	−16 (−69, 37)	−2 (−61, 57)
**Do not take off PPEs during cleaning waste related vehicles every day**	10 (2.88)	103 (30)	27 (22, 32)	13 (8.55)	12 (8)	−0.5 (−6, 6)	**27 (19, 35)**	**26 (17, 35)**

#### 3.3.2 Observed practice

The intervention appears to have improved the handwashing practices of the workers before wearing PPEs (DID: 27%, CI: 2, 52), after finishing work (DID: 31%, CI: −7, 69) and during work intervals (DID: 30%, CI: −33, 93). However, it did not improve the adherence to proper handwashing protocols (DID: 22%, CI: −42, 86) and handwashing practices before having snacks/meals (DID: 2%, CI: −61, 65), and after using the toilet (DID: 7%, CI: −41, 55). Workers' improved practices of maintaining COVID-19 prevention measures such as coughing etiquette (DID: 20%, CI: 0.2, 40), not sharing while smoking or drinking (DID: 11%, CI: −27, 49), maintaining social distance at the workplace (DID: 13%, CI: −19, 46), handwashing before touching face (DID: 31%, CI: 8, 54) were more likely to have positive associations with the intervention. while, although improved in endline intervention areas, the practice of mask use (DID: 1%, CI: −40, 42) was less likely to be associated. The intervention might have improved the practices of cleaning equipment after work (DID: 9%, CI: −8, 26) but did not improve the practice of disinfecting the equipment (DID: −14%, CI: −34, 6) by the workers. Also, the observed practices of the workers in keeping waste in designated places seemed to be motivated by the intervention (DID: 9%, CI: −8, 26) (Table 5).

**Table 5:** Observed practice of the workers in maintaining OHS and COVID-19 prevention measures.

Indicators	Intervention			Control			DID (CI)
	Baseline n (%) N = 32	Endline n (%) N = 35	Difference (CI)	Baseline n (%) N = 15	Endline n (%) N = 15	Difference (CI)	
Washed hands	N = 5	N = 17		N = 11	N = 6		
Before Wearing PPE	0 (0)	3 (18)	18 (−0.4, 35)	1 (9.09)	0 (0)	−9 (−26, 7)	**27 (2, 52)**
After wearing PPE	1 (20)	4 (24)	4 (−36, 44)	2 (18)	0 (0)	−18 (−40, 4)	22 (−24, 68)
After finishing work	4 (80)	16 (94)	14 (−13, 41)	11 (100)	5 (83)	−17 (−43, 10)	31 (−7, 69)
During intervals of work	3 (60)	12 (71)	11 (−37, 58)	4 (36)	1 (17)	−19 (−61, 21)	30 (−33, 93)
Before having snacks/meals	2 (40)	12 (71)	31 (−17, 78)	6 (55)	5 (83)	28 (−13, 71)	2 (−61, 65)
After using toilet/urinate	1 (20)	3 (18)	−2 (−36, 32)	1 (9.09)	0 (0)	−9 (−43, 25)	7 (−41, 55)
**Washed hands following proper handwashing steps**	2 (40)	6 (35)	−5 (−50, 40)	3 (27)	0 (0)	−27 (−72, 18)	22 (−42, 86)
**COVID precautions observed**							
Wear face mask	13 (41)	24 (69)	28 (5, 51)	7 (47)	11 (73)	27 (−7, 61)	1 (−40, 42)
Wear goggles	0 (0)	3 (8.57)	8 (−1, 17)	0 (0)	1 (6.67)	6 (−8, 20)	2 (−15, 19)
Used elbow/cloth while sizing/coughing	2 (6.25)	7 (20)	14 (−1, 29)	1 (6.67)	0 (0)	−6 (−18, 6)	**20 (0.2, 40)**
Didn't share cigarettes/drinking cup	16 (50)	12 (34)	−16 (−39, 7)	6 (40)	2 (13)	−27 (−56, 3)	11 (−27, 49)
Maintained social distance	7 (22)	10 (29)	7 (−11, 25)	1 (6.67)	0 (0)	−6 (−33, 21)	13 (−19, 46)
Washed or sanitized hands before touching face, mouth, eye	0 (0)	4 (11)	11 (0.8, 21)	3 (20)	0 (0)	−20 (−40, 0.2)	**31 (8, 54)**
**Organized at work**							
Kept equipment organized	29 (91)	31 (89)	−2 (−16, 12)	12 (80)	12 (80)	0 (−28, 28)	−2 (−34, 30)
Throw waste in designated places	31 (97)	35 (100)	3 (−6, 12)	14 (93)	13 (87)	−6 (−20, 8)	9 (−8, 26)
Washed equipment after work	20 (63)	22 (63)	0 (−23, 23)	13 (87)	12 (80)	−7 (−33, 19)	7 (−28, 42)
Disinfect equipment after work	5 (16)	3 (8.57)	−7 (−22, 8)	0 (0)	1 (6.67)	7 (−5, 19)	−14 (−34, 6)
Wash gum boot after work	6 (19)	11 (31)	12 (−8, 32)	3 (20)	7 (47)	27 (−5, 59)	−15 (−53, 23)

## 4 Suggestions to improve OHS situation

The main demands from the workers were to increase their OHS-related measures in both baseline and endline. These included getting free medical facilities (Intervention-baseline: 55%, endline: 74%; control-baseline: 49%, endline: 56%) or health insurance (Intervention-baseline: 16%, endline: 27%; control-baseline: 3.95%, endline: 15%), receiving a regular and increased supply of PPEs (Intervention-baseline: 35%, endline: 43%; control-baseline: 46%, endline: 45%), and having the opportunity to use upgraded waste and sanitation management technologies (Intervention-baseline: 23%, endline: 20%; control-baseline: 20%, endline: 23%) (Table 6)

**Table 6:** Opinion of the workers on improving their OHS status.

Indicators	Intervention			Control			DID (CI)
	Baseline n (%) N = 347	Endline n (%) N = 349	Difference (CI)	Baseline n (%) N = 152	Endline n (%) N = 150	Difference (CI)	
Upgrade technology	79 (23)	69 (20)	−3 (−9, 3)	31 (20)	35 (23)	3 (−6, 12)	−6 (−17, 5)
Health insurance	54 (16)	93 (27)	11 (5, 17)	6 (3.95)	23 (15)	11 (5, 17)	0 (−9, 9)
Free medical facility	190 (55)	259 (74)	19 (12, 26)	75 (49)	84 (56)	6 (−5, 17)	13 (−0.4, 26)
Increase and maintain the PPE supply	123 (35)	150 (43)	8 (0.3, 14)	70 (46)	67 (45)	−1 (−12, 10)	9 (−4, 22)

## 5 Discussion

We conducted the study to understand the effect of an occupational health safety intervention on the waste and sanitation workers in Bangladesh. We found that the intervention improved workers' knowledge of communicable diseases, including the key COVID-19 transmission and prevention components, their understanding of the importance of using and maintaining PPEs, and workers' attitudes toward using and washing PPEs and work equipment. The intervention also improved workers' practice of regularly changing/cleaning masks and equipment, maintaining hygiene after returning home from work, handwashing before-during-after work, maintaining coughing etiquette and social distancing at the workplace, and disposing of waste in designated areas. The intervention also ensured the availability of necessary PPEs to the workers and successfully impacted the use of those diverse PPEs. On the other hand, the intervention was less effective in improving workers' understanding of social distancing, quarantine, and isolation, their attitude towards handwashing before and after using toilets and having meals, and their practice of disposing of PPEs, handwashing before taking food and after using the toilet, using the mask, and disinfecting equipment.

The intervention made the occupational health and safety guidelines available to the workers in the intervention areas. It ensured that the workers understood what the guideline contained, which may have impacted the improved adherence to guidelines in intervention areas more than in control areas. It may have impacted the workers' knowledge, attitude, and practices.

To improve the worker's occupational health and safety, context-specific and appropriate intervention, especially training, has been recommended in different LMIC settings, including Bangladesh (Alam, 2021, Asibey et al., 2019, Degavi et al., 2021, Gebremedhin et al., 2016, Repon et al., 2015, Tsukiji et al, 2020, World Bank et al., 2019). Interventions are recommended to be low-cost and implemented considering the context-specific occupational hazards that need to be addressed (Emmatty et al., 2019). To reduce COVID-19 exposure and related occupational hazards, the International Labour Organization (ILO) suggested training provisions on properly using and disposing of PPEs by waste workers and regularly communicating with them about COVID-19 situation updates and mitigation strategies (Papandrea, 2020).

The intervention from ITN-BUET was designed so that the workers receive training from the people they know or work with, as it might have a better chance of meeting the workers' contextual demands. This initiative also reduced the chance of potentially spreading misinformation, which was largely a concern during COVID-19 (Organization, 2020). The intervention also used more pictorials and visualizations so that the workers, regardless of their literacy status, could easily grasp the meaning, which might also have contributed to the impact. Improvement in workers' understanding of COVID-19 transmission and preventive measures suggests that even after massive and intensive mass media interventions, interpersonal communication that directly addresses the workers, such as training, can significantly improve the workers' knowledge of important OHS issues. On the other hand, the workers' understanding of mask use and correctly knowing COVID-19-related terms such as social distancing, quarantine, and isolation have not changed much as the workers from both intervention and control areas were already similarly exposed to these issues. During the COVID-19 pandemic, massive mass media interventions across the globe focused on COVID-19 and the importance of mask use (Bakebillah et al., 2021, Srivastava et al., 2020), and it seems like the workers already had a better understanding of those. Thus, there was no noticeable difference in this aspect. Nonetheless, the mass media interventions also mainly focused on improving people's knowledge of COVID-19 transmission and preventive measures, which improved even further among the waste and sanitation workers after the intervention.

Workers from the intervention areas could also easily perceive the benefit of the training compared to workers in control areas indicating the appropriate reach of the intervention to the workers. The learnings from the training events might have improved their attitude (in intervention areas) towards the importance of using and washing PPEs, cleaning waste management tools regularly, and injury management. On the other hand, the workers' attitude towards handwashing before critical handwashing time, such as before meals and after using the toilet, did not show any association with the intervention. Different development agencies and the government of Bangladesh have been working on improving people's handwashing, including waste and sanitation workers, for many years, and previous large-scale trials observed low improvement in handwashing with soap practices (Wichaidit et al., 2019). Thus, it is unsurprising that this short training period did not improve the workers' handwashing (with soap) attitude before meals or after defecation. Future interventions need to find additional techniques (in addition to this intervention) to address the issue of handwashing with soap.

During COVID-19, the family members of the waste and sanitation workers were particularly in danger of getting infected (Alam, 2021, Alam et al., 2022, WaterAid, 2020, WaterAid Bangladesh, 2020)]. The knowledge dissemination at the training session seems to have helped the workers improve their practices (reported) of taking measures to keep their families safe from COVID-19. Despite not changing their handwashing attitude during key times, the workers began to wash their hands immediately after returning from work to keep their families safe in intervention areas.

Fear arousal was one of the primary incentives for people to maintain protective conduct among threat perception indicators during CVOID-19 (Sand et al., 2022). Hence, it can be argued that fear of COVID-19 might have impacted the worker's decision more significantly than the impact of the intervention, thus, it will not be applicable in non-COVID-19 contexts. However, Both control and intervention groups were impacted by the fear of COVID-19, and we measured the changes (which is considered as the effect of the intervention) based on the difference in changes from intervention areas to control areas, thus allowing the interpretation to be more intervention effect specific. For instance, in the case of the use of PPEs, we have seen that it was improved by 6% in both intervention and control areas; although it was improved in the intervention areas, we can't attribute this as an intervention effect as similar improvement was also seen in the areas without intervention (control areas). On the other hand, the practice of keeping physical distance from the family members before cleaning (when the workers return from work) improved in both intervention areas and control areas. However, in intervention areas, the improvement was significantly larger (32%) than it was in control areas (10%), indicating a clear impact of the intervention. In that case, COVID-19 fear might have caused a 10% improvement in both intervention and control areas, but the remaining 22% improvement in the intervention areas can be attributed to the effect of the intervention.

Ensuring the availability of appropriate PPEs (including consideration of gender appropriateness) to the waste and sanitation workers was recommended as a crucial measure to reduce occupational health risks, including COVID-19 infection and transmission (PAHO, 2020, Papandrea, 2020). The intervention increased the availability of different PPEs such as masks, hand sanitizer, gloves, goggles, gumboot, regular shoes, and reflective jackets to the workers in intervention areas rather than in control areas, and the workers' started wearing those PPEs in intervention areas. However, the workers in the control areas also wore the PPEs that were available to them then. This practice in both intervention and control areas indicates that the availability of PPEs ensures its use in many cases. In Nepal, the unavailability of PPEs was the major driver for the waste workers' inadequate PPE-wearing practices (Adhikari et al., 2021). Thus, it can be inferred that future intervention needs to continue providing PPEs and ensuring regular supply to the workers. Moreover, the workers' common complaints about the PPEs they received in intervention areas, including the inappropriate fitting of the PPEs, suffocation, and feeling of heat and sweating, need to be addressed in the future. Dissatisfaction about PPEs and the irregularity of their use among waste and sanitation workers were reported to be caused by their lack of comfortability in PPE design, particularly when the hot climate of tropical areas is not considered. Also, PPEs are often purchased in bulk without regard for the workers' fitting issues (Somani and Hueso, 2020). Future interventions must consider providing comfortable PPEs and ensuring their regular supply to the workers. More intensive research is required to produce contextually appropriate PPEs.

Changing behaviour is a complex process and it is normal to experience many lapses and relapses when trying to change behavior because it can be an inherently unstable and unsteady process (Alam et al., 2023, Bouton, 2014). In order to improve waste management and OHS behaviour, previous researchers have emphasized training as a crucial intervention element (Joshi et al., 2015, Tudor et al., 2005). However, to have a sustainable behaviour change among the workers, regular monitoring from the municapility office, and provision of continuous intervention might be needed.

The study had a few limitations. We conducted the study just one month after the intervention provision to see the immediate effect. However, the impact of the intervention might be different after six months or a year, which can be explored in the future to realize the long-term impact of the intervention. Also, we selected the study sites (both intervention and control areas) purposively; thus, it does not fully represent the situation of the sanitation workers in the country. Nonetheless, we selected the sites from all eight divisions, considered the cultural and geographical diversities, and ensured representation from large cities (city corporations) and small towns (municipalities). Moreover, during observation of the workers' practices at the workplace, the workers did not perform all activities at all times, resulting in a few observations in certain aspects. Also, many sanitation workers (especially drain cleaners and septic tank/pit emptiers) were working late at night, making it harder for the researchers to reach the workers at the workplace because of security concerns and the unavailability of safe transportation. These affected the number of observed practices.

## 6 Conclusion and recommendations

The study aimed at evaluating the effect of the OHS intervention on the waste and sanitation workers and identify the areas of improvement. The study found that the intervention was successful in improving the critical measures such as knowledge about spread and prevention of infectious disease and key COVID-19 prevention strategies, attitude towards using PPEs, and practice of taking preventive measures after they return home from work to ensure occupational health safety of the waste and sanitation workers. This intervention can be adapted for other cities of Bangladesh and other low-middle income country settings with additional actions taken to make the intervention context-appropriate.

Based on the endline evaluation findings, we recommend the followings actions.

• Organize refresher training for workers and their supervisors at least once a year and orientation events following the health safety guidelines for the new workers on maintaining occupational health and safety.
• In future interventions, address occupational health-related injuries and health complications such as joint or back pain with regular health checkups/insurance.
• Create a balance between the quality and comfort of the PPEs, especially for the safety of gumboots in future designs, and create a mechanism to maintain an adequate supply of these once damaged.
• Workers should be encouraged to be responsible for using PPEs regularly through on-job training and supervision.
• A detailed study and intervention should be considered to address the occupational health safety problems of the workers holistically. These may include improved salary structure, stable job contracts, decent workplaces, insurance, medical facilities, better education and training facilities, improved accommodation, WASH access, and proper protective equipment use.

## Author contributions

DMS and TA with MA prepared the initial draft of the manuscript. KFT, MH, FS, MR, MF, MAR, AA, and JBT reviewed the study design, tools for data collection, and the manuscript. DMS, MH, FS, and KFT monitored the data collection activity. KFT and MH conducted data cleaning and analysis. MF, MR, DMS, MA and TA was involved in data analysis and provided intellectual input. All authors thoroughly reviewed the manuscript and provided scientific input. MA supervised the entire study.

## Funding and acknowledgments

This research study was funded by the Bill and Melinda Gates Foundation (BMGF) and administered by the Centre for Water Supply and Waste Management of International Training Network of Bangladesh University of Engineering and Technology (ITN-BUET) - (Grant/Award Number INV-007345 to ITN-BUET). icddr,b acknowledges with gratitude the commitment of BMGF to its research efforts. icddr,b is also grateful to the Governments of Bangladesh, and Canada for providing core/unrestricted support.

## Declaration of Competing interest

The authors declare no conflicts of interest.
